# Partitioning no-take marine reserve (NTMR) and benthic habitat effects on density of small and large-bodied tropical wrasses

**DOI:** 10.1371/journal.pone.0188515

**Published:** 2017-12-07

**Authors:** Garry R. Russ, Jake R. Lowe, Justin R. Rizzari, Brock J. Bergseth, Angel C. Alcala

**Affiliations:** 1 College of Science and Engineering, James Cook University, Townsville, Queensland, Australia; 2 Australian Research Council Centre of Excellence for Coral Reef Studies, James Cook University, Townsville, Queensland, Australia; 3 Fisheries and Aquaculture Centre, Institute for Marine and Antarctic Studies, University of Tasmania, Hobart, Tasmania, Australia; 4 Silliman University Angelo King Center for Research and Environmental Management (SUAKCREM), Dumaguete City, Negros, Philippines; Department of Agriculture and Water Resources, AUSTRALIA

## Abstract

No-take marine reserves (NTMRs) are increasingly implemented for fisheries management and biodiversity conservation. Yet, assessing NTMR effectiveness depends on partitioning the effects of NTMR protection and benthic habitat on protected species. Such partitioning is often difficult, since most studies lack well-designed sampling programs (i.e. Before-After-Control-Impact-Pair designs) spanning long-term time scales. Spanning 31 years, this study quantifies the effects of NTMR protection and changes to benthic habitat on the density of tropical wrasses (F. Labridae) at Sumilon and Apo Islands, Philippines. Five species of wrasse were studied: two species of large-bodied (40–50 cm TL) *Hemigymnus* that were vulnerable to fishing, and three species of small-bodied (10–25 cm TL) *Thalassoma* and *Cirrhilabrus* that were not targeted by fishing. NTMR protection had no measurable effect on wrasse density, irrespective of species or body size, over 20 (Sumilon) and 31 (Apo) years of protection. However, the density of wrasses was often affected strongly by benthic cover. *Hemigymnus spp*. had a positive association with hard coral cover, while *Thalassoma spp*. and *Cirrhilabrus spp*. had strong positive associations with cover of rubble and dead substratum. These associations were most apparent after environmental disturbances (typhoons, coral bleaching, crown of thorns starfish (COTS) outbreaks, use of explosives and drive nets) reduced live hard coral cover and increased cover of rubble, dead substratum and sand. Disturbances that reduced hard coral cover often reduced the density of *Hemigymnus spp*. and increased the density of *Thalassoma spp*. and *Cirrhilabrus spp*. rapidly (1–2 years). As hard coral recovered, density of *Hemigymnus spp*. often increased while density of *Thalassoma spp*. and *Cirrhilabrus spp*. often decreased, often on scales of 5–10 years. This study demonstrates that wrasse population density was influenced more by changes to benthic cover than by protection from fishing.

## Introduction

Coral reef ecosystems are under increasing threat from both natural and anthropogenic environmental disturbances[1–3], and appear particularly vulnerable to global warming [2, 3], increased frequency and intensity of storms [4, 5], declining water quality [4, 6] and overfishing [7]. These disturbances often modify processes and structure in the benthic habitat, with subsequent effects on reef fish populations [8–10]. For example, severe environmental disturbances such as typhoons and coral bleaching reduce coral cover, and often decrease structural complexity and coral diversity [4, 9, 11]. Conversely, less intense environmental disturbances may only result in minor changes to the benthos and thus reef fish assemblages [12]. Variation in type, severity and frequency of environmental disturbance events, and the specific benthic preferences of the reef fish, will determine the extent of response in density of coral reef fishes. For example, live coral-dependant species (e.g. corallivores) are negatively impacted by coral loss caused by environmental disturbances to the greatest degree [8, 13–15]. However, not all reef fish will decline in density following environmental disturbances to the benthos [11, 16, 17]. For example, parrotfish and goatfish that often feed over dead surfaces (sand, rubble, dead coral), commonly increase in density following shifts in benthic cover from live coral to dead surfaces [11, 16, 17]. These changes in reef fish density can be sustained for years to decades, but eventually fish populations return to pre-disturbance states as hard coral recovers [8–10]. While some reef fish increase in local density due to disturbance events, it is currently accepted that environmental disturbances to the benthos affect density of more reef fish groups negatively than positively [11, 14, 18].

While many studies have documented changes to benthos and fish assemblages following environmental disturbances, few have focussed on species that may benefit from disturbance-mediated coral loss [11, 14, 19]. Moreover, the paucity of appropriate sampling designs (e.g. Before-After-Control-Impact-Pair designs) monitored on decadal scales has meant that many studies are temporally limited, often focusing on a single environmental disturbance over a 1–3 year timescale [9, 11, 20]. Consequently, few studies have documented long-term post-disturbance recovery of coral and fish assemblages [10, 21], or the impacts of multiple environmental disturbance events on fish and benthic assemblages [22, 23] and subsequent recovery from such events [8, 13–15]. Our understanding of how changes to benthic habitat affect the densities of different reef fish taxa on decadal-scales represents a significant knowledge gap in coral-reef ecology.

Fishing pressure is another important driver of density of reef-fish populations, and in extreme cases can cause the collapse of entire fish stocks [24, 25]. No-take marine reserves (NTMRs) are increasingly implemented to achieve sustainable fisheries management and conservation of targeted species. While NTMRs can increase body size, density and biomass of fishery-targeted species [23, 26–28], research has documented differential effects of protection on other moderately-targeted and non-targeted species, including declines in fish populations [26, 27]. These differential responses are well documented [9, 11, 12], and have a number of possible causes including the intensity and targeting of fishing pressure [29], variable recruitment in time and space [29, 30], variable availability of resources (i.e. benthic habitat) [31, 32], environmental stochasticity [33], fishing methods [27, 34], NTMR size and proximity to humans [27], site history [35] and the compliance with NTMR regulations [27, 36]. Furthermore, the effects of NTMR protection on non-targeted species remain to be fully elucidated [37]. Non-target species typically do not respond directly to NTMR protection, but may respond to indirect effects of NTMR protection that often take decades to accrue [10, 21]. For instance, non-target species may be indirectly affected if they are the prey or competitors of species that do respond positively (i.e. increase in biomass or density) to NTMR protection [21], or they may have a density response if the NTMR affects the quality of benthic habitat [38]. Factors influencing the density responses of coral-reef fishes to NTMR protection can vary greatly among, and within, taxonomic groups [26] and can become particularly complex for targeted demersal fishes with a strong affinity to particular benthic habitats, such as wrasses.

Wrasses (F. Labridae) are benthic and planktivorous feeding reef fishes that are common on coral reefs [39]. Based on ecological characteristics and targeting of fishing, wrasses investigated here can be split into two main groups: small-bodied, non-target wrasses (*Thalassoma hardwickii*, *T*. *lunare* and *Cirrhilabrus spp*.*)* and large-bodied, targeted wrasses (*Hemigymnus melapterus* and *H*. *fasciatus)*. Species within each group were chosen due to their relatively high abundance at the study sites. *Thalassoma* comprises 27 species, which are broadly distributed throughout the Indo-Pacific and Atlantic regions [40]. Like most wrasses, they possess protruding caniniform teeth which are used to feed primarily on benthic invertebrates, fish eggs [39, 41, 42], and occasionally small fish [43]. *Thalassoma*. *hardwickii* and *T*. *lunare* grow to a maximum of 18cm and 25cm TL, respectively [43]. *Thalassoma* are found across the majority of reef habitats [41, 44, 45]. *Cirrhilabrus spp*. are small planktivorous wrasses that inhabit reefs (up to 90m deep) throughout the Pacific [46]. They have small conical teeth that aid in capturing zooplankton, which forms their main food source [47]. *Cirrhilabrus spp*. can grow to 11cm TL and are common inhabitants of the reef flat [48]. *Hemigymnus melapterus* and *H*. *fasciatus* are large-bodied benthic carnivores that inhabit coral and rocky reefs throughout the Indo-Pacific region [43, 49]. *H*. *melapterus* and *H*. *fasciatus* are reported to grow to 90cm and 80cm in length, respectively, yet most fish rarely exceed 50 cm TL [43]. No commercial fishery exists for these two large wrasses in developed nations, but they are likely captured incidentally in recreational hook and line fisheries. In developing regions of the tropical Indo-Pacific, these large wrasses are commonly captured by spear, trap, hook and line and gill nets in subsistence fisheries [25, 50, 51].

Wrasses, like many reef fish, tend to be species-specific in terms of their response to NTMR protection [50, 52, 53]. Different body sizes, diets, vulnerabilities to fishing and habitat preferences among wrasses result in different rates and patterns of recovery of fish density in NTMRs [21, 53, 54]. Determining the characteristics of particular wrasses that make them respond to NTMR protection or benthic change in certain ways may be useful to reef managers. In terms of NTMR protection, small-bodied wrasses are not usually fishery targets in developed or developing nations, and thus are not expected to directly benefit from NTMR protection. Yet throughout the tropics, large-bodied wrasses are commonly harvested as primary and secondary targets of reef fisheries and thus these larger-bodied wrasses might be expected to benefit from NTMR protection in developing nations where fishing pressure can be moderate to high [51, 55, 56].

This study examines how wrasses are affected by changes to benthic habitat and NTMR protection at two small NTMR’s in the central Philippines. Apo and Sumilon NTMRs were first established in 1982 and 1974, respectively. Apo has had 31 years of continuous and effective protection. Sumilon reserve has had a complex history of protection including management breakdowns and pulse fishing events (see S1 Table). The fish assemblages of the Philippines have been subjected to major environmental disturbance events which have resulted in substantial declines in coral cover in past decades [16, 17, 57]. Consequently, due to the history of benthic change and a history of long-term NTMR protection, these sites are particularly useful for partitioning the relative importance of NTMR protection and benthic change on the density of tropical wrasses. This study addresses two questions: (1) What are the long-term effects of benthic habitat changes on the density and assemblage structure of Philippine wrasses that differ in body size and thus vulnerability to fishing?; and (2) How does long-term NTMR protection affect the density and assemblage structure of both moderately-targeted and non-targeted Philippine wrasses?

## Materials and methods

### Ethics statement

No animals were captured, manipulated or sacrificed during this study and no protected species were sampled. No specific permits for sampling were required for this study as all data were obtained via observational underwater visual fish counts on scuba. NTMR sites were protected from fishing by local fishing communities and all sites were positioned on public land.

### Study sites

**Fig 1:**
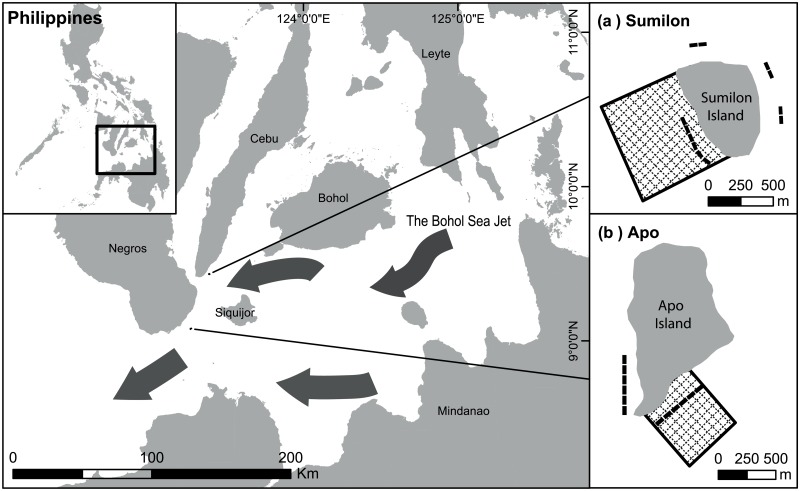
Location of the study sites in the central Philippines. Inset A: Sumilon Island. Inset B: Apo Island. Crosshatching indicates no-take marine reserve area. Black rectangles show approximate positions of permanent 50 m * 20 m replicate transects for fish and benthic surveys.

This study was conducted at Apo (9°4’N, 123° 16’E) and Sumilon Islands (9°21’N, 123°23’E) in the central Philippines (Fig 1). NTMR status was relatively well maintained by local fishing communities during the investigation, with some breakdowns of NTMR protection at Sumilon reserve in the 1980s and 1990s. Each of the two islands had a “fished” and a “NTMR protected” site. These four sites were monitored for both benthic composition and reef fish density almost annually (26 times in 31 years) from 1983 to 2014 by a single observer (GRR). Sumilon and Apo NTMRs were both affected by coral bleaching in 1998. Apo NTMR was impacted in 2010 by a local storm and again in 2011 and 2012 by strong typhoons. Both Apo and Sumilon Islands were exposed to intense fishing pressure (traps, hook and line, spear, gill nets) prior to the implementation of NTMRs [35]. Apo NTMR has an excellent history of protection and compliance since its implementation in 1982 (31 yrs.). Sumilon NTMR has a complex history of protection (see S1 Table).

### Surveys of fish density

Six replicate underwater visual censuses (UVC) were performed almost annually in both NTMR and fished sites at Apo and Sumilon Islands. The 50m * 20m (1000m^2^) replicate UVCs estimated wrasse density on the reef slope (3-17m in NTMRs, 9-17m in fished areas) in November/December of each sampling year. Only fish > 5 cm TL were counted. These sampling methods (i.e. location of replicates, census method, time of year and observer (GRR)) were consistent for the 31 years of study (1983–2014). However, to account for the high density of some small-bodied wrasses Log4 abundance categories (Category 1 = 1 fish, Category 2 = 2–4 fish, Category 3 = 5–16 fish, Category 4 = 17–64 fish, Category 5 = 65–256 fish, Category 6 = 257–1024 fish, Category 7 = 1025–4096 fish, Category 8 = 4097–16384 fish) were used to estimate density of *Thalassoma spp*. for the first ≈15 years of the study *(T*. *hardwickii* 1983–1999, *T*. *lunar*e 1983–2000). Actual counts of individual fish per replicate were made for these species from 1999 for *T*. *hardwickii* and from 2000 for *T*. *lunare*, onward. The Log4 abundance categories were used to estimate density of *Cirrhilabrus spp*. for the entire study. Density of *Hemigymnus spp*. was based on actual counts of individuals per replicate.

The Log4 abundance categories (1–8) were converted to “best estimates” of actual density in different ways for *Thalassoma spp*. and *Cirrhilabrus spp*. For *Thalassoma spp*., actual count data from 1999 to 2014 were placed into a Log4 abundance category. The mode of the frequency distribution of actual counts within an abundance category for the period 1999 to 2014 was used as the best estimate of actual density in a Log4 abundance category for the period 1983–1998 for *T*. *harwickii* and 1983–1999 for *T*. *lunare*, respectively. These “best estimates” of density for *T*. *hardwickii* were Category 1 = 1 fish, 2 = 2, 3 = 5, 4 = 17, 5 = 180, 6 = 600, 7 = 1200, 8 = 4097, and for *T*. *lunare* Category 1 = 1 fish, 2 = 2, 3 = 10, 4 = 25, 5 = 100, 6 = 257, 7 = 1026, 8 = 4097. For *Cirrhilabrus spp*., “best estimates” of density within an abundance category were either the mid-point of the category (Categories 1–6 = 1, 3, 10, 40, 160, 640 fish, respectively) or the minimum within that category (Categories 7 and 8 = 1025 and 4097 fish, respectively).

### Surveys of benthos

Benthic cover was estimated from 1983–1999 via line intercept transects in the same location as the fish surveys. These transects were performed directly after the fish surveys. Benthic cover was recorded every 20cm along a 50m transect tape. There were 6–9 replicate transects per site for all fished and NTMR sites at Apo and Sumilon Islands. Major benthic categories were: (1) branching and tabular coral (CBCT) (2) massive and encrusting coral (CMCE), (3) soft coral (SC), (4) rubble (R), (5) sand (S), (6) hard dead substratum (HDS), (7) macroalgae and (8) other. A structural complexity index (SCI) was estimated for each transect, on a scale of 0–4, with 0 = flat and 4 = highly complex.

Post-1999, benthic cover and composition was obtained by subdividing each 50m*20m fish transect into ten 10m*10m quadrats. Benthic cover was then estimated within each quadrat visually to within approximately 5% accuracy. Mean benthic cover was calculated for each transect (50m * 20m) by averaging data from the ten quadrats. For subsequent statistical analyses, all dead substratum components (R, S and HDS) were grouped to form a single “dead substratum” (DS) category.

Polynomials were fitted to fish density and hard coral cover to emphasize trends over time at each site, in particular to highlight where major changes to coral cover led to major changes in fish density.

### Data analysis

In order to account for changes in the sampling techniques for wrasse density, *Thalasssoma spp*. densities were split into two separate time periods. These were 1983–1998 and 1999–2014 for *T*. *hardwickii* and 1983–1999 and 2000–2014 for *T*. *lunare*. These time periods were subsequently treated separately in all univariate data analyses, to avoid confounding results due to differences in density estimation techniques.

Generalised additive mixed models (GAMMs), with a poisson error distribution, were used to partition the effects of NTMR protection and benthic habitat on wrasse density. GAMMs were chosen because they accounted for the possibility of nonlinear relationships between response and predictor variables [58]. Predictor variables included effects of NTMR status, time (duration of protection), and benthic variables (i.e. CBCT, CMCE, SC, DS, SCI) on wrasse density. Separate GAMMs were performed at Apo and Sumilon Islands for each of the fish groups/species (i.e. *Hemigymnus melapterus*, *H*. *fasciatus*, *Thalassoma hardwickii*, *T*. *lunare*, *and Cirrhilabrus spp*.). NTMR status, time, and benthic variables were treated as fixed effects, while replicate transects were treated as a random effect. GAMMs were fitted using the *mgcv* package in R [59]. Model selection for GAMMs was based on minimization of the Akaike Information Criterion corrected for small sample sizes (AICc) [60]. The smallest AICc value identified the model with the greatest support. Relative support for one model was determined by calculating the differences between its AICc and the smallest AICc (ΔAICc) and scaling these differences into model weights (*w*AICc). All models with values of ΔAICc ≤ 2 are presented, since values within this threshold can have similar explanatory power [60]. It should be noted that while not all models are presented, model weights were calculated using AICc values from all models considered in the analysis (i.e. starting with a global model with all predictor variables mentioned above). Detection of a positive NTMR effect required two things. Firstly, an increase in fish density within the NTMR relative to the fished control site over time. Secondly, an important NTMR Status * Time (duration of protection) interaction, where important is defined as the interaction appearing in any GAMM model within 2 AIC values of the top model for each species at each location.

Non-metric multidimensional scaling (nMDS) performed in PRIMER v6 [61], was used to examine assemblage structure of wrasses. These nMDS were conducted using Bray-Curtis dissimilarities and square-root transformed data, which were chosen to down-weigh abundant species. Structure of the benthos was also assessed with nMDS, using Euclidean distances on data that were normalised and natural log-transformed in order to increase spread. At each location the six replicate transects per site per year were averaged and analysed with mean fish density per species. Benthic surveys were not conducted at Apo Island and Sumilon fished sites from 1988 to 1992, thus the corresponding data were not included in analysis.

## Results

### NTMR effects on labrid density

**Fig 2:**
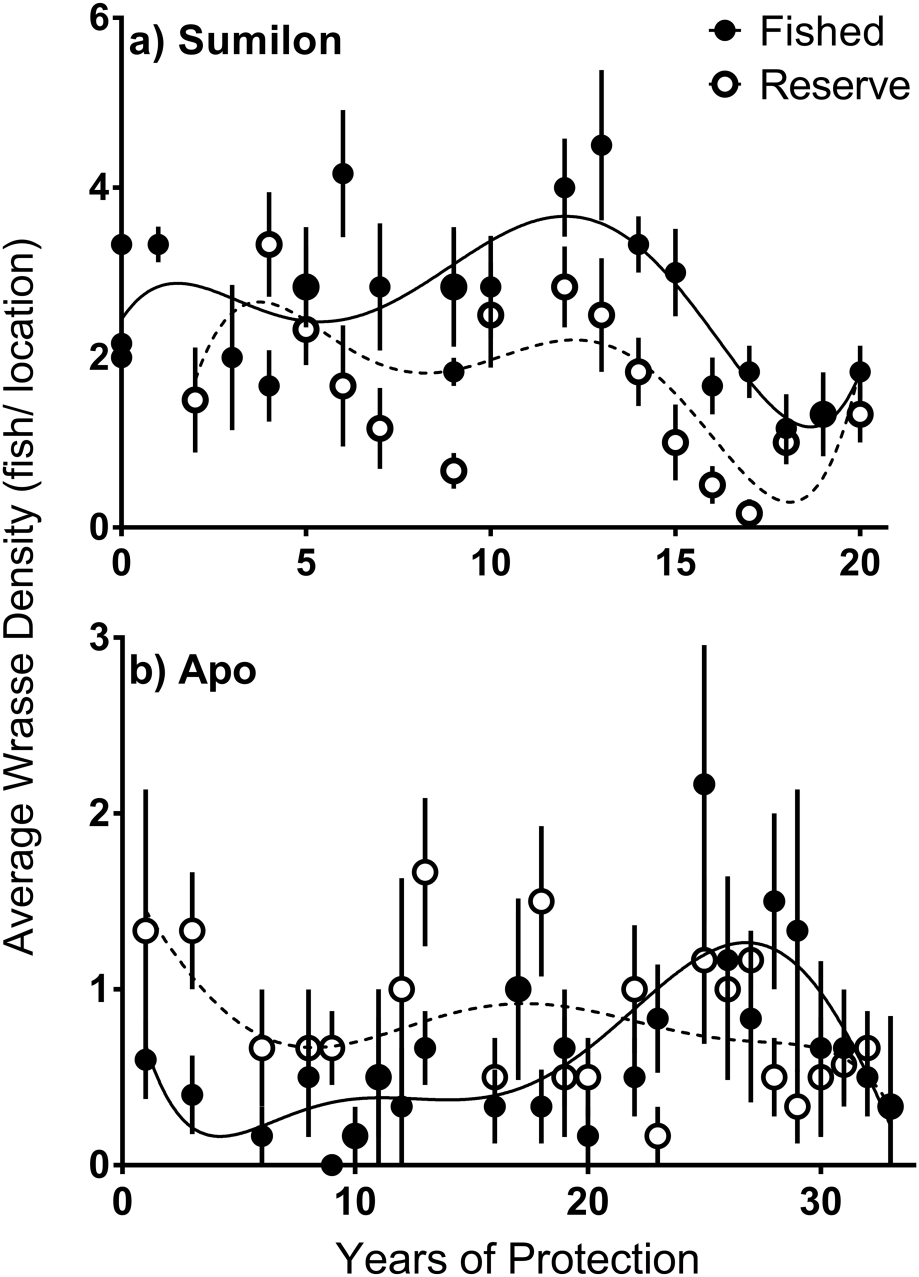
Long-term (1983–2014) trends of *H*. *melapterus* density. a) Sumilon NTMR and fished sites, b) Apo NTMR and fished sites. Error bars are standard error (SE). Trend lines are polynomials.

**Fig 3:**
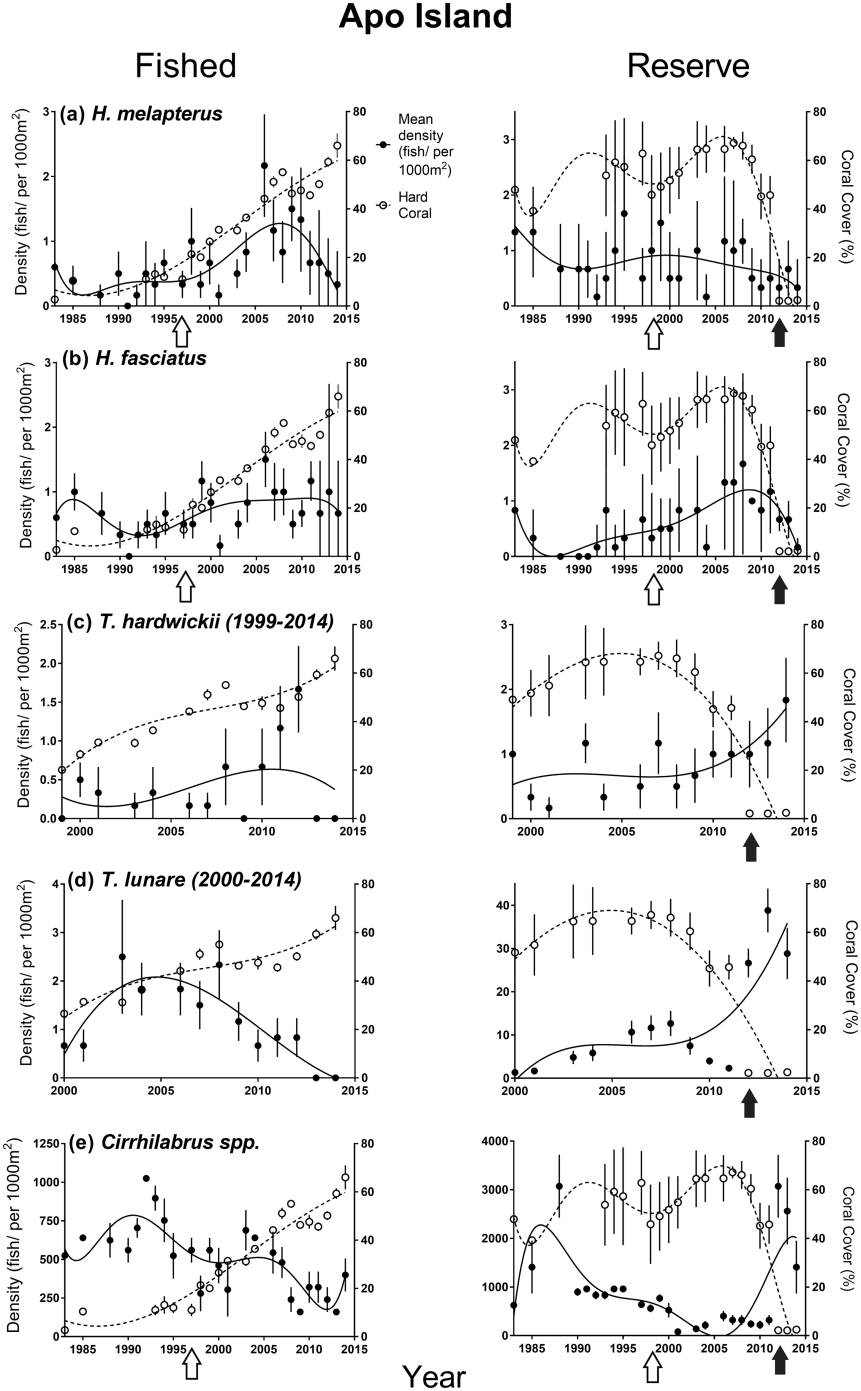
Long-term temporal trends in density of (a) *H*. *melapterus*, (b) *H*. *fasciatus*, (c) *T*. *hardwickii* (1999–2014), (d) *T*. *lunare* (2000–2014), (e) *Cirrhilabrus spp*., and temporal trends in cover of hard corals (CBCT and CMCE combined) at Apo fished and NTMR (reserve) sites. Wrasse density is shown by black points and continuous trend lines. Hard coral cover is shown by white data points and dotted trend lines. Environmental disturbance events are indicated by arrows: 1998 coral bleaching event–white, 2011 and 2012 typhoon events–black. Standard errors (SE) are displayed on plots, along with polynomial trend lines.

**Fig 4:**
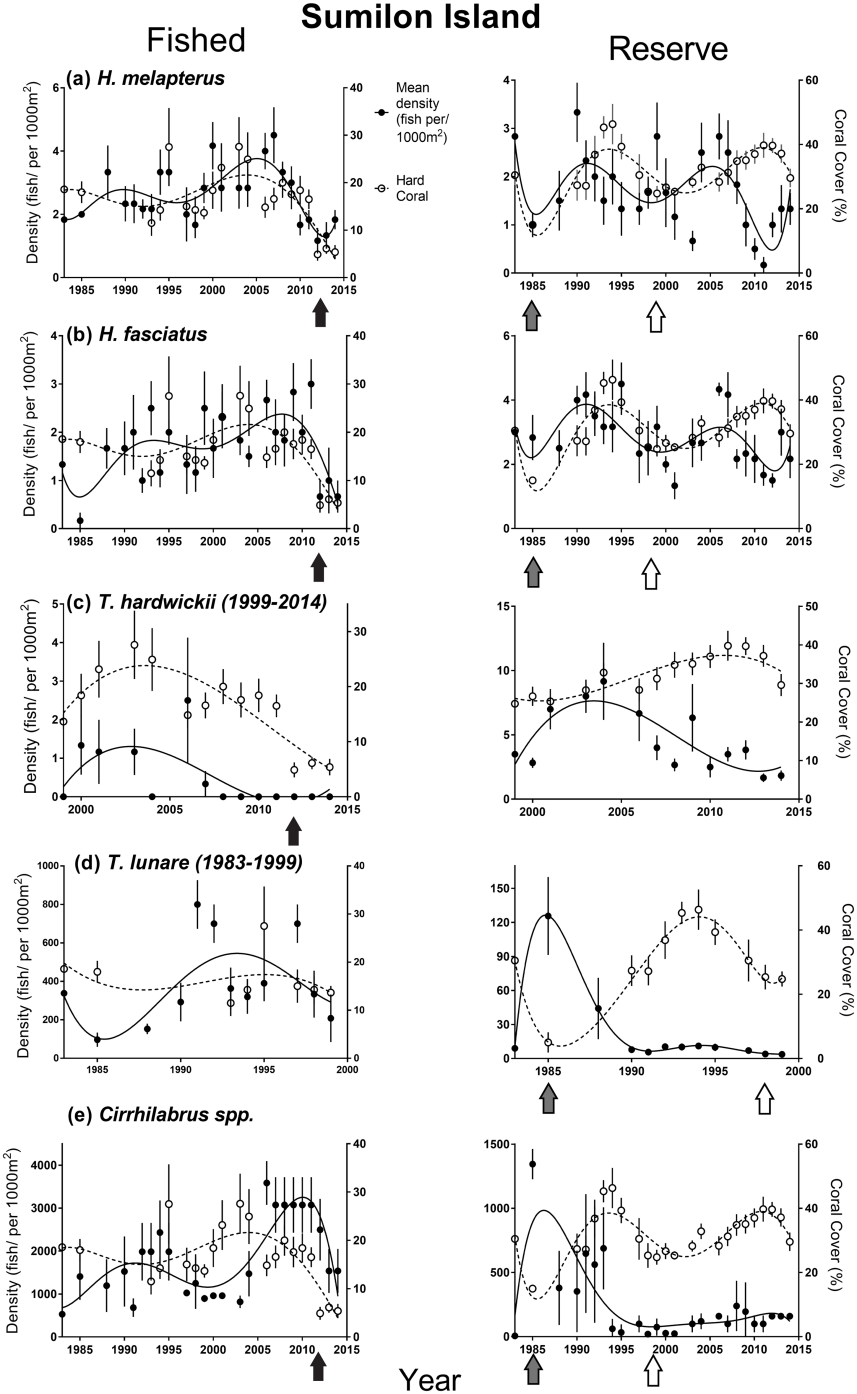
Long-term temporal trends in density of (a) *H*. *melapterus*, (b) *H*. *fasciatus*, (c) *T*. *hardwickii* (1999–2014), (d) *T*. *lunare* (1983–1999), (e) *Cirrhilabrus* spp., and temporal trends in cover of hard corals (CBCT and CMCE combined) at Sumilon fished and NTMR (reserve) sites. Wrasse density is shown by black points and continuous trend lines. Hard coral cover is shown by white data points and dotted trend lines. Environmental disturbance events are indicated by arrows: 1984 to 1986 destructive fishing event–grey, 1998 coral bleaching event–white, 2012 typhoon event–black. Standard errors (SE) are displayed on plots, along with polynomial trend lines.

Fifteen of thirty-nine NTMR Status * Time interactions were identified as important from the GAMMs (Table 1). However, we did not detect any increases or decreases in fish density in reserves relative to fished areas consistent with a NTMR effect. One example of a lack of NTMR effect is *Hemigymnus melapterus*, a large-bodied, targeted wrasse (Fig 2). At Apo, just three of eleven important NTMR Status * Time interactions were detected for the small non-target wrasses (*T*. *hardwickii*, *T*. *lunare* and *Cirrhilabrus spp*.*)*, reflecting patterns of fish density over time that were also not consistent with a NTMR effect (Table 1, Fig 3). A similar result was obtained in the GAMMs for density of most wrasse species at Sumilon Island (Table 1, Fig 4), but no species displayed a pattern of change in fish density over time consistent with a NTMR effect. Rather, the GAMMs indicated that benthic habitat was often an important driver of wrasse density. The frequent effects of benthic habitat variables on wrasse density were relatively consistent at both sites (Table 1). The larger-bodied labrids (*H*. *melapterus* and *H*. *fasciatus)* had an affinity for hard coral habitat, particularly branching and tabular coral morphologies, while the smaller non-target wrasses (*T*. *hardwickii*, *T*. *lunare* and *Cirrhilabrus spp*.*)* preferred dead substratum as a benthic habitat (Table 1, Figs 3 and 4).

**Table 1:** Optimal generalized additive mixed models (GAMMs) estimating effects of NTMR status, time (duration of protection) and cover of benthos on wrasse density at (a) Apo and (b) Sumilon Islands.

Location	Model	df	LogLik	AICc	ΔAICc	wAICc
**(a) Apo**						
*H*. *melapterus*	**Status + Time + DS + SC**	8	-382.46	781.60	0.00	0.21
	Time + CBCT	5	-386.08	782.40	0.87	0.14
	Status * Time	5	-386.12	782.50	0.95	0.13
	Time + SC + CBCT	7	-384.45	783.40	1.87	0.08
*H*. *fasciatus*	Time * Status + CBCT	8	-276.38	569.40	0.00	0.15
	CBCT	5	-279.88	570.00	0.62	0.11
	Status + CBCT	6	-279.33	571.00	1.63	0.07
*T*. *hardwickii*						
*1983–1998*	**CBCT**	4	-142.31	293.30	0.00	0.53
	DS	4	-143.28	295.20	1.93	0.20
*1999–2014*	**Status + Time + CMCE**	7	-216.73	448.20	0.00	0.16
	Status + Time	5	-219.08	448.50	0.37	0.14
	Status*Time	6	-218.76	450.00	1.89	0.06
	Status*Time + CMCE	8	-216.62	450.10	1.99	0.06
*T*. *lunare*						
*1983–1999*	**Status + Time + CBCT + CMCE +DS + SC +SCI**	14	-221.91	480.10	0.00	0.53
	Time + CBCT + CMCE + DS + SC + SCI	13	-223.66	480.30	0.27	0.47
*2000–2014*	Status*Time + DS	8	-518.88	1054.70	0.00	0.24
	DS + SCI	7	-520.48	1055.70	1.00	0.14
*Cirrhilabrus spp*.	Stats*Time + CBCT + CMCE +DS + SC +SCI	15	-23671.51	47375.20	0.00	1.00
**(b) Sumilon**						
*H*. *melapterus*	**Status + Time + CMCE + DS**	8	-253.53	523.70	0.00	0.16
	Status*Time + DS	7	-254.76	524.00	0.34	0.135
	Status + Time + DS	6	-255.84	524.00	0.38	0.13
	Status + Time + CMCE	6	-256.30	524.90	1.29	0.08
	Status*Time + CMCE + DS	9	-253.45	525.70	1.99	0.06
*H*. *fasciatus*	**Status + Time**	4	-240.89	490.00	0.00	0.17
	Status*Time	5	-239.90	490.10	0.10	0.17
	Status + Time + CMCE	6	-239.00	490.40	0.40	0.14
	Status	3	-242.67	491.40	1.49	0.08
*T*. *hardwickii*						
*1983–1998*	**Status + SCI**	6	-224.63	462.40	0.00	0.12
	Status + DS	6	-224.63	462.40	0.00	0.12
	Status + Time	5	-225.82	462.50	0.04	0.11
	Status*Time	6	-224.84	462.80	0.42	0.09
	Status + Time + SCI	7	-223.71	463.00	0.56	0.09
	Status + Time +DS	7	-223.71	463.00	0.56	0.09
	Status*Time + SCI	8	-223.19	464.40	2.00	0.04
	Status*Time + DS	8	-223.19	464.40	2.00	0.04
*1999–2014*	**Status + Time + CBCT + DS**	8	-329.04	675.00	0.00	0.85
*T*. *lunare*						
*1983–1999*	Status*Time + CMCE +DS + SC + SCI	13	-549.63	1130.80	0.00	1.00
*2000–2014*	Status*Time + CBCT + CMCE + DS + SC +SCI	15	-768.34	1569.80	0.00	1.00
*Cirrhilabrus spp*.	Status*Time + CBCT + CMCE + DS + SC +SCI	15	-34550.23	69132.50	0.00	1.00

Substratum types are: CBCT = Coral Branching + Coral tabulate, CMCE = Coral Massive + Coral Encrusting, DS = dead substratum, SCI = structural complexity, SC = soft coral. Top-ranked model is displayed in bold.

### Effects of environmental disturbances on benthos

Throughout the 31-year study, both Apo and Sumilon islands were affected by a number of environmental disturbances (Figs 3 and 4). Between 1983 and 1985, an illegal breakdown in reserve protection at Sumilon NTMR led to a use of destructive fishing techniques (explosives, drive nets) that reduced hard coral cover by 50%, and substantially increased cover of dead substratum (sand, rubble and hard dead substratum)(Fig 4). Coral bleaching in 1998 reduced hard coral cover and increased cover of dead substratum at two of the four study sites (Apo NTMR and Sumilon NTMR) (Figs 3 and 4). The reduction of hard coral cover at Sumilon NTMR in 1998 was also associated with a COTS outbreak. Coral cover increased substantially for ≈10 years in both reserves following the 1998 bleaching event (Figs 3b and 4b). At the Apo fished site, the 1998 bleaching event killed soft corals which had been the dominant benthic cover for the previous 15 years [13]. This bleaching event caused a marked shift at the Apo fished site from a soft coral-dominated benthos to one dominated by hard corals (mostly *Acropora*), with hard coral cover increasing from 10% in 1998 to 60% in 2014 (Fig 3a). Hard coral cover again declined substantially within the Apo NTMR when a storm (2010) and two typhoons (2011 and 2012) impacted the benthos (Fig 3a). These major disturbance events resulted in hard coral cover dropping in the Apo NTMR from ≈60% to <5% from 2009 to 2012–2014 (Fig 3a). After the storm and typhoons, the reef slope of the Apo NTMR was dominated by dead substratum (coral rubble, hard dead substratum and sand). The Sumilon fished area was also impacted by the same typhoon in 2012, reducing hard coral cover from 20% to < 5% (Fig 4a).

### Effects of environmental disturbances on density of wrasses

Responses of wrasses to environmental disturbances were species-specific, but relatively consistent among sites. In general, *Hemigymnus* density was positively related to hard coral cover in the majority of the top GAMM models at each island, while density of *Thalassoma* and *Cirrhilabrus spp*. was positively correlated with dead substratum at each island (Table 1, Figs 3 and 4).

*Hemigymnus melapterus* increased in density at the Apo fished site for 14 years (1997–2010) as hard coral cover, specifically branching and tabular corals, increased substantially over the same period. This increase in hard coral cover at the Apo fished site was due to the bleaching event in 1998 that caused a shift in dominance from soft coral to branching and tabular hard coral (Fig 3a). *H*. *melapterus* density then declined at the Apo fished site for unknown reasons. *H*. *melapterus* maintained density at Apo NTMR from 1983 to 2009 and then declined in density following the storm in 2010 and two typhoons in 2011 and 2012 that reduced coral cover substantially (Fig 3a). *H*. *melapterus* density had a positive relationship with hard coral cover at the Sumilon fished site for 31 years, and declined substantially after the 2012 typhoon decreased live coral cover (Fig 4a). At the Sumilon NTMR, *H*. *melapterus* density declined sharply between 1983 and 1985 during the destructive fishing event (Fig 4a). Hard coral cover also declined sharply between 1983 and 1985 at Sumilon NTMR, then recovered from 1987 to 1994 (Fig 4a). *H*. *melapterus* density at Sumilon NTMR increased as coral cover increased from 1987 to the mid-1990s, declined after the 1998 bleaching and crown of thorns starfish event, and then increased again from 2003–2007 as coral cover increased (Fig 4a). *H*. *melapterus* density declined from 2008–2011 in the Sumilon NTMR, despite coral cover being high, before recovering from 2012–2014 (Fig 4a). *H*. *fasciatus* densities also had a positive relationship with hard coral cover (Table 1, Figs 3b and 4b). *H*. *fasciatus* density increased at the Apo fished site from 1991 to 2006 and then maintained density until 2014 (Fig 3b). *H*. *fasciatus* density increased at the Apo NTMR from 1991 to 2011 as hard coral cover increased (Fig 3b) but then declined sharply in 2012 to 2014, as hard coral cover declined sharply after the two typhoons (Fig 3b). *H*. *fasciatus* density mirrored cover of hard coral closely at the Sumilon fished site for most of the study, with density decreasing sharply from 2012 to 2014 as hard coral cover declined due to the 2012 typhoon (Fig 4b). At the Sumilon NTMR, *H*. *fasciatus* density increased from 1985 to 1995 as coral recovered after the destructive fishing event, declined during the coral bleaching event in 1998, then recovered from 2001 onwards as coral cover increased again, declining for unknown reasons from 2007–2012 (Fig 4b).

Density of *Thalassoma* and *Cirrihilabrus spp*. were, for the most part, positively associated with cover of dead substratum. Density of *Thalassoma hardwickii* and *T*. *lunare* increased sharply at the Apo NTMR from 2012 to 2014 after cover of hard coral declined due to the typhoons in 2011 and 2012 (Fig 3c and 3d). *T*. *lunare* density declined for over a decade at the Apo fished site (2000–2014) as cover of hard coral increased (Fig 3d), but such a pattern was not clear for *T*. *hardwickii* at the same site over the same period (Fig 3d). *T*. *hardwickii* declined in density from 2003 to 2014 at Sumilon NTMR as coral cover increased (Fig 4c), but such a pattern was not as clear at the Sumilon fished site (Fig 4c). *T*. *lunare* increased in density sharply at Sumilon NTMR between 1983 and 1985 as coral cover declined sharply due to use of explosives and drive nets (Fig 4d), then declined to very low density from 1990 to 2000 as hard coral cover increased in Sumilon NTMR (Fig 4d). A negative relationship between density and coral cover was not as clear for *T*. *lunare* at the Sumilon fished site (Fig 4d). Hard coral cover had a clear negative effect on *T*. *lunare* and *T*. *hardwickii* density in the majority of GAMM models at each island (Table 1, Figs 3 and 4). Density of *Cirrhilabrus spp*., after being relatively low for almost 15 years (1997–2011), increased substantially and rapidly in the Apo NTMR from 2012 to 2014 after hard coral cover declined sharply due to the typhoons in 2011 and 2012 (Fig 3e). Density of *Cirrhilabrus spp*. declined for almost two decades at the Apo fished site (1994–2014) as cover of hard coral increased (Fig 3e). The decrease in density of *Cirrhilabrus spp*. at the Sumilon fished site from 2011 to 2014 following the typhoon was likely due to the fact that this disturbance caused the reef slope to be covered in sand (which is a less preferred benthic substratum for this genus), rather than coral rubble (Fig 4e). At the Sumilon NTMR, density of *Cirrhilabrus spp*. increased sharply between 1983 and 1985 following a sharp decline in coral cover due to use of explosives and drive nets (Fig 4e), then declined from 1985–1994 as coral recovered, remaining at relatively low density from 1995–2014 as coral cover remained high (Fig 4e). Cover of dead substratum had a positive effect on *Cirrhilabrus spp*. density in most GAMM models at each island (Table 1, Figs 3 and 4).

### Assemblage structure of benthos and wrasses among sites and over time

**Fig 5:**
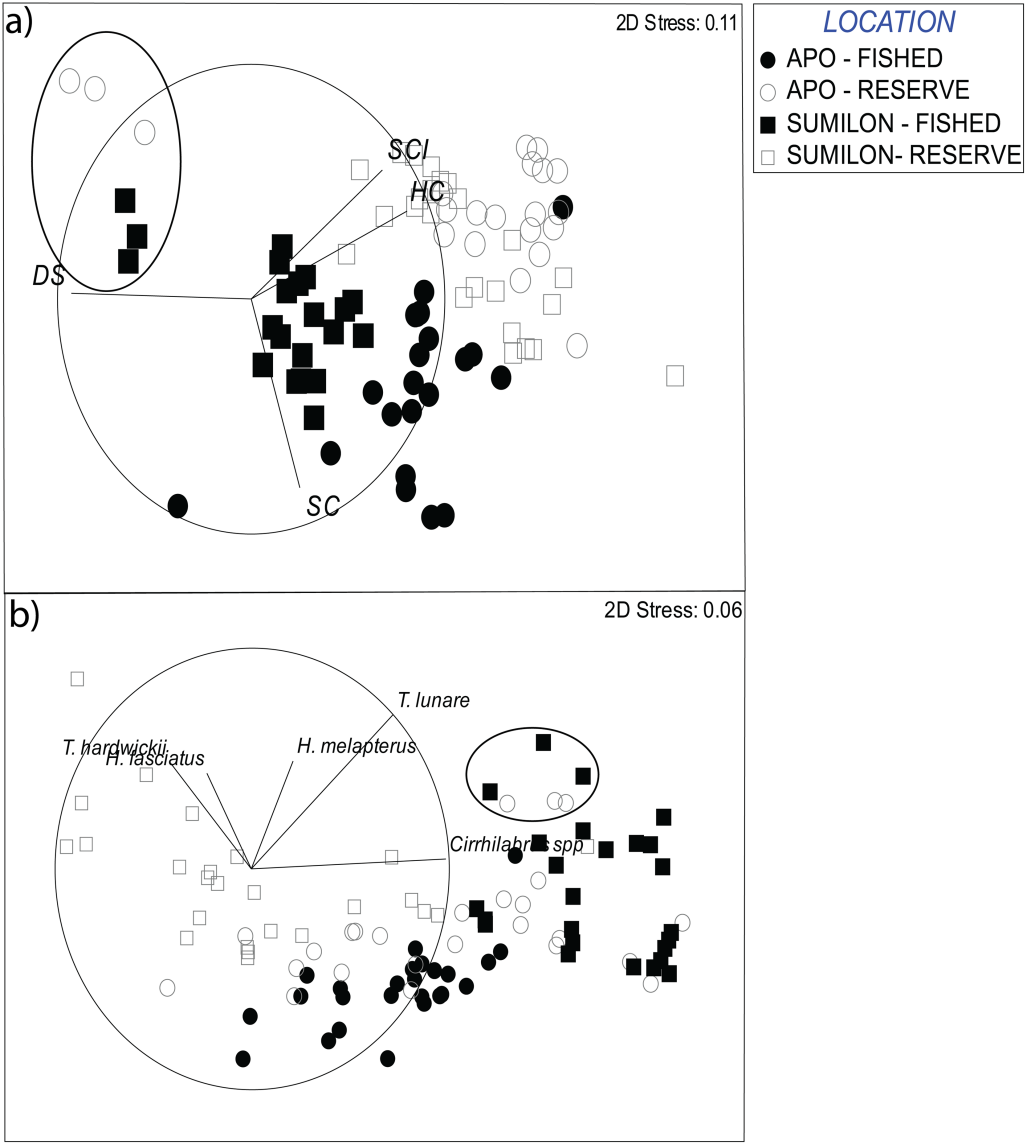
Non-metric multidimensional scaling (nMDS) performed on distance matrices of benthic cover and wrasse assemblage structure. a) Assemblage structure of the benthos at Sumilon and Apo Islands, b) assemblage structure of wrasses at Sumilon and Apo Islands. Black shapes represent fished sites, white shapes represent NTMR sites. Benthic components include: DS = dead substratum, HC = hard coral (CBCT and CMCE combined), SC = soft coral, SCI = structural complexity. Assemblage shifts from HC to DS dominance post-2011 and2012 typhoon events at Apo NTMR and Sumilon fished sites are indicated by an eclipse.

Assemblage structure of the benthos differed among the four sites at the outset, and these differences remained relatively distinct over the 31 years of study (Fig 5a). NTMRs were characterized by high percentage cover of hard corals and high structural complexity, while fished sites were characterized by high percentage cover of dead substrata and soft corals (Fig 5a). Assemblage structure of the benthos was very consistent over time in the two NTMRs, with the exception of Apo NTMR and Sumilon fished site in 2012 to 2014 following the two typhoons (Fig 5a). Assemblage structure of the benthos at the Apo fished site was not as clustered in multidimensional space as the other three sites, reflecting the long-term change in dominant benthos from soft corals (1983–1998) to hard corals (1999–2014) at this site (Fig 5a). The distinct benthic assemblages among sites and over time resulted in distinct assemblage structure of wrasses among sites for 31 years (Fig 5b). Fished sites, with higher cover of dead substratum, were characterized by high density of *Cirrhilabrus spp*. and *Thalassoma lunare* (Fig 5b). NTMRs tended to have higher coral cover and higher density of *Hemigymnus fasciatus* and *Thalassoma hardwickii*. (Fig 5b).

## Discussion

This study demonstrates the importance of benthic habitat as a determinant of wrasse density. Three key findings stand out: 1) Densities of large-bodied wrasses targeted by fishing were not affected by NTMR protection; 2) benthic composition drove densities of wrasses in the long-term; 3) benthic composition drove assemblage structure of wrasses. The strong relationship between benthic composition and density of tropical wrasses has previously been described [8, 14, 15], however this investigation is unique in that the influences of benthic composition on targeted and non-targeted Philippine wrasses were examined in a 31 year “natural experiment” that included multiple environmental disturbances to the benthos, and long-term recovery of the benthos from these disturbances.

The lack of an even moderate NTMR effect on the larger-bodied *H*. *melapterus* and *H*. *fasciatus* at either NTMR was unexpected, but is consistent with reports for *H*. *melapterus* at Sumilon and Apo NTMRs for the first 13 and 25 years of protection, respectively [21]. It is possible that this lack of a direct NTMR effect on the density of these large-bodied wrasses may have been due to an indirect effect of increased density of large predatory reef fish in these NTMRs (12). However, availability of potential prey for predators had a larger influence than the effect of potential predators on prey at these two NTMRs (12). Even for these large-bodied wrasses, which are subject to an intermediate level of fishing pressure [62], benthic habitat was an important driver of density and assemblage structure. Increases in hard coral cover generally resulted in higher densities of *Hemigymnus*. This is a slightly unexpected result considering that the diet of *Hemigymnus* is benthic invertebrates and that there are high densities of benthic invertebrates found on degraded reefs [63, 64]. Yet, positive habitat associations between corals and *Hemigymnus spp*. have previously been observed [65, 66]. Recent research suggests that the genus *Hemigymnus* has developed increased bite force, velocity and jaw protrusion as functional adaptations to the challenges of capturing diverse prey types in shallow water environments [67, 68]. Therefore, it is possible that *Hemigymnus* are adapted to feeding upon invertebrates found commonly in structurally complex, high coral cover habitats of coral reefs.

The strong relationship between benthic habitat and wrasse density was evident, irrespective of species, body size, diet or vulnerability to fishing. Habitat effects outweighed the effects of NTMR protection for all species examined. This result was consistent with previous studies for smaller-bodied non-target wrasses (*T*. *lunare*, *T*. *hardwickii* and *Cirrhilabrus spp*.)[8, 9]. In the Philippines these small, non-target species are subject to low fishing pressure and thus do not respond to decreased fishing effort that NTMRs provide [51].

The present study suggests that larger-bodied species of wrasse (*H*. *melapterus* and *H*. *fasciatus)* were, for the most part, positively associated with hard coral cover, and, for the most part, smaller-bodied species (*T*. *hardwickii*, *T*. *lunare* and *Cirrhilabrus spp*.*)* were negatively correlated with hard coral cover. Other studies have reached similar conclusions [8, 9, 69], but the present study was able to differentiate between the effects of benthic habitat change and fishing pressure on local wrasse densities at two NTMRs and two control, fished sites. This 31 year “natural experiment” included multiple environmental disturbance events, providing long-term trends in wrasse density that could be related to changes in benthic cover. Temporal trends in wrasse density were related to temporal trends in live hard coral cover for a number of reasons. First, preliminary data investigation indicated differential responses of wrasses to particular benthic components, such as sand, hard dead substratum and rubble that were grouped into one category “dead substratum” for presentation and analysis. The “dead substratum” benthic category is most characteristic of disturbed coral reef environments. Second, the majority of the coral reef literature that reports on environmental disturbances generally concentrates on the loss of live hard coral cover and its effects, rather than the increase in cover of dead substratum [8, 9, 11]. The present study has an added advantage, in that it documents long-term (5–10 year scale) recovery of benthos and fish. Thus, this study concentrated on loss and recovery of live hard coral and has been presented in a way as to be consistent with the majority of literature on environmental disturbances to coral reefs.

Assemblage structure of benthos and wrasses were spatially distinct among sites from the beginning of the study, and these differences remained consistent over 31 years. NTMRs were placed on the side of each island with a steep coralline wall with high coral cover, whereas “fished” areas were placed on the opposite side of each island, where the reef slope was more gradual and the benthos was dominated by sand, rubble and low hard coral cover [10, 16]. The different benthos among sites largely explains the differences in wrasse assemblage structure among sites. This confounding of NTMR protection and benthic habitat was largely unavoidable. Thus, from the outset, NTMR sites had higher slope, hard coral cover and structural complexity than fished sites.

An increasing number of studies have addressed the importance of partitioning the effects of benthic habitat and fishing on abundance and biomass of coral-reef fishes [10, 20, 70, 71]. However, partitioning these effects over large time scales is still rare [8, 9, 11, 14, 15, 72]. Previous attempts to partition the effects of benthic habitat and fishing pressure on the density of tropical wrasses have been limited in temporal extent and often have grouped wrasse species together [73–76]. When wrasses have been investigated at a level of genus or species [8, 9, 21] wrasse densities appear to link strongly with disturbance-mediated coral loss [8, 9, 71]. To date, the literature suggests that many species of tropical wrasse are habitat generalists [8, 9]. Furthermore, apart from very clear declines in density of obligate corallivores (e.g. *Labrichthys unilineatus)*, the density responses of tropical wrasses to coral loss caused by environmental disturbances does not appear to be strongly linked to dietary preference [8, 9, 71]. For example, a meta–analysis has reported different density responses to environmental disturbances for two closely related tropical wrasse with similar diets: *Hemigymnus melapterus* and *H*. *fasciatus* [9]. Thus, it is advantageous to examine density responses to coral loss by wrasses at a species or functional level.

Decreases in density of the larger-bodied, moderately-targeted wrasses (*Hemigymnus spp*.) following declines in coral cover may have reflected emigration from unsuitable to suitable habitat rather than mortality. This response is common in larger-bodied species following disturbances that have resulted in substantial coral loss [9, 11, 16, 17] and in this case is likely a product of the characteristically large home range of *Hemigymnus*, which can extend hundreds of meters [77, 78]. Larger-bodied species often require additional resources [79, 80] and are often more efficient in translocating from place to place than small-bodied fishes [80–85], making emigration from unfavourable to favourable habitats less taxing for larger-bodied species and thus more common [80, 84]. This large home range could also have contributed to the lack of an NTMR effect on *Hemigymnus* density in this study. The marked increases in density of smaller-bodied, non-target wrasses (*Thalassoma spp*., *Cirrhilabrus spp*.) after declines in coral cover are likely explained by both the short-term migration of adults to areas of dead substratum (favoured benthic habitat), and longer-term recruitment of juveniles into these same areas. The smaller-bodied wrasses displayed rapid increases in density after environmental disturbances resulted in decreased hard coral cover, particularly following the 2011 and 2012 typhoons at the Apo NTMR and Sumilon fished site. Previous studies which have documented the increases in density of small, non-target species that commonly follow large-scale disturbance events throughout the Pacific, have also suggested that mass immigration is the likely driver of short-term population increase [11, 69, 86].

The long-term (decadal) correlation between hard coral cover and density of *Hemigymnus spp*. is likely caused by real changes in local population density, rather than any short-term migration effect. While environmental disturbances increase density of small-bodied wrasses in the short term, long-term trends suggest that these changes to local populations of small-bodied wrasses represent real changes in population density that are maintained only while suitable benthic habitat remains. As coral recovers, these smaller-bodied labrids likely translocate to preferred habitat nearby. The population dynamics of large–bodied, moderately-targeted wrasses and small non-target wrasses are likely linked to habitat associations. Some variation is expected between wrasses that differ in habitat preference, for example wrasses associated with live hard coral would be expected to show opposite changes in density in response to environmental disturbances to those wrasses preferring dead substratum. More information on habitat selection of wrasses at settlement would be beneficial [87] to differentiate migration from settlement and survival processes.

Our results indicate that wrasse densities on coral reefs in the central Philippines are affected more by benthic habitat change than protection from fishing in NTMRs. For large-bodied wrasses these results are somewhat surprising, given the intermediate harvest levels that these wrasses are subject to in the central Philippines. Live hard coral cover was the strongest driver of wrasse density, with smaller-bodied *Thalassoma* and *Cirrhilabrus spp*. associated with dead substratum, while large-bodied *Hemigymnus spp*. were associated with live hard coral cover. Any increase in severity and frequency of environmental disturbances [9, 11, 15] could have substantial consequences for the large and small-bodied wrasses investigated in this study. Micro-invertivores such as tropical wrasses, are suggested to have a low extinction risk from fishing but a moderate extinction risk from environmental disturbances related to climate change [71]. Our findings support these suggestions for the large-bodied wrasses, *Hemigymnus melapterus* and *H*. *fasciatus*, which declined in density following coral loss and were not substantially impacted by fishing pressure. Conversely, environmental disturbances that reduced live hard coral cover leads to increases in the density of small-bodied *Thalassoma spp*. and *Cirrhilabrus spp*. These small-bodied wrasses may thus become more common components of benthic fish assemblages as coral cover and reef health declines. The long-term (decadal) nature of the present study demonstrates the importance of benthic composition, both inside and outside NTMRs, as a primary driver of wrasse density.

## Supporting information

S1 Table Protection history of study sites, 1974–2014. White bars = years that sites were open to fishing, grey bars = years when sites were open to hook and line fishing, and black bars = years when sites were closed to fishing. Asterisks = years of sampling.
(XLSX)
